# Supplementary material for: Automatic detection of cell-cycle stages using recurrent neural networks
Source: PLoS One. 2024 Mar 11;19(3):e0297356. doi: 10.1371/journal.pone.0297356 (PMC10927108; doi:10.1371/journal.pone.0297356)
Supplement: Attachment — Submitted filename: Rebuttal_letter.pdf [file pone.0297356.s001.pdf]

Response to the reviewers.
We thank the reviewers for their critical assessment of our approach. In the following, we address the major concerns raised point by point, and the corresponding revisions.

Comments from Reviewer 1

Comment: 1. Did the authors use any statistical methods at any points in the manuscript? Please update and include the details in the respective statistical analysis section.

Reply: Thanks for your comment. Kindly note that the reported numbers in the paper are the average performance of 20 repetitions and we have included the standard deviations of each experiment as well in the tables. Please check line number 447.

Comment: 2. Whether the change of hyperparameters will have a drastic effect on the model's performance is hoped that the authors can prove it experimentally.

Reply: Thanks for the comment. The results reported have already undergone grid search-based hyperparameter tuning. The optimum hyperparameters selected by the grid search are provided in Table 1, and the standard deviation of 20 repetitions is already mentioned in the paper. Please check line 447 and 448 in the manuscript.

Comment: 3. From the performance of label matrix, the superiority of the model proposed by authors seems not obvious, and it is hoped that this results can be further explained.

Reply: It is true that visually finding the differences in the label matrix can be challenging. To address this, we have increased the size of the label matrix plots to make the differences more clear. Please refer to Fig. 6, Fig. 7, Fig. 10, Fig. 11, and Fig. 13 for visual representations. Additionally, we have included quantitative metrics such as precision, recall, and F-score. To further illustrate the advantages of incorporating time information, we have included the PCA plots. Furthermore, the accuracy, which is a qualitative measure of the label matrix, is clearly demonstrated in the accuracy table, highlighting the advantages of the proposed method. We have further added the following sentence in the results section. "The label matrix generated by the ResNet18 model contains some misclassifications in the post-mitosis class and also in the mitosis class for some sequences which is eliminated by the proposed RNN-based model. The same behaviour is observed for the other datasets as well which is shown in the respective subsections." (Please refer to line numbers 552 to line 555 in the manuscript).

Comment: 4. The baseline proposed by authors is relatively few, and it is hoped that some baseline other than RNN-based can be added.

Reply: Kindly note that, we have included the ResNet18 model which is not an RNN-based approach for comparison. We have also compared our model with LiveCellMiner paper and the baseline model we have developed. We are confident that the proposed baseline reflects the state-of-the-art.

Comments from Reviewer 2

Comment: 1. The accuracies, precisions, recalls, and F-scores showed in Table 3, 5, 7, 9, and 11 did not prove the consistent superior performance of Time Encoded ResNet18. In Table 11, the model reached the top F-score in only 2/6 states, which showed that in some cases, the model could not even outperform the base model. Since the evaluation criteria were quite close among four methods (close to 1.000), I didn't see the advantage of Time Encoded ResNet18.

Reply: Kindly note that, even though the results for the Inter, Prometa, Meta, and Telo phases are somewhat lower, they are quite close to those of the best-performing model. In the two states where the results are better, we observe that the numbers are significantly higher compared to the second best-performing model. Also please refer to the Tables 3, 7, and 9 in the results section. It is quite clear that the results are only lower for mitosis state and for the other two states, the F-score is already higher.

Comment: 2. Fig. 13 shows the predictions of each method and the true labels. But I can hardly tell which method was better. Authors may compare the key difference of each method and plot the figures focusing on the difference.

Reply: Thanks for pointing out this. It is true that label matrix plots were small. Now we have increased the size of the plots and updated the label matrix plots and the latent space plots. Kindly refer to the response to comment 3 of reviewer 1 in the rebuttal letter.

Minor comments:

Comment: 3. Please define the term "sequence" clearly.

Reply: Kindly note that a "Sequence" typically refers to a series of related or connected frames. We have indicated that now in the introduction. Please refer to line 13 in the manuscript.

Comment: 4. Please explain why models treat each image independently in line 423.

Reply: The image is treated independently here as it is a classifier model and time information is not used. Please refer to line 422 in the manuscript.

Comment: 5. The typo in equation 8.

Reply: We have now corrected the typo in equation 8. Please refer to line 418.

Comments from Reviewer 3

Comment: 1. In their literature review, the authors mentioned a state-of-the-art methods to identify cell-cycle stages, CellCognition and LiveCellMiner. However, they did not compare the performance of their model with CellCognition and only compared with LiverCellMiner on the "LiveCellMiner Dataset". In my point of view, other competing methods they adopted were more like an ablation study. Therefore, I suggest adding some experiments to compare the performance of their Time Encoded ResNet18 with CellCognition and LiveCellMiner on both the "LiveCellMiner Dataset" and the "Zhong et al.'s Dataset".

Reply: CellCognition is an annotation tool used for finding complex cellular dynamics. It uses a whole-slide 2D+t image which contains multiple cells to detect objects and classify them into cell-cycle stages. However, in our case, both the datasets are single cell datasets which contain only one cell. Since our focus is on single cell image datasets, it is not reasonable to compare with CellCognition. Furthermore, the Zhong et al.'s dataset even uses CellCognition for object detection and feature extraction from the whole-slide images. Kindly note that, the comparison results of the performance of the Time Encoded ResNet18 with LiveCellMiner and TC3 are already available.

Comment: 2. In their experiments, the training and testing were performed within the same dataset. However, in practice, after getting some new time-lapse microscopy images, it is usually time-consuming to annotate some of them to get a training set. Therefore, I suggest the authors to test the performance of their model when training on one dataset, say, the LSM710 dataset and predicting the cell-cycle stages of another dataset containing the same type of cells (maybe after fine-tuning on a small fraction of data), say, the LSD1 dataset (also capturing the human HeLa cells). Or, maybe they can test their model on one dataset they used but with a smaller train-test ratio, say, 0.5 or even 0.2. I think these settings will make their model more attractive and helpful for users.

Reply: Thanks for the comment. We guess the performance will drop quite a bit if we change the modality. The proposed experiment of checking what fraction of ground truth is needed to obtain good results would, however, be quite interesting and also a hint for potential users how many images they should annotate to obtain good results. We have done the experiment for train test ratio of 0.5 and the results are now updated in Table 10. It shows that the numbers do not drop significantly indicating that even with a smaller dataset, the model is able to give good performance.

Comment: 3. It seems that the improvements in accuracy, recall and F-score were quite marginal in some experiments, for example, Table 2, Table 8 and Table 10. Also, I noticed that in several settings, their Time Encoded ResNet18 had a lower F-score than LiveCellMiner for the identification of cells belonging to the mitosis stage (Table 3, Table 7, Table 9). Therefore, I was wondering if the improvements in classification brought by their model can help obtain some biological findings. For example, the authors of LiveCellMiner [1] analyzed the NikonXLight dataset and found that downregulation of INO80, SRCAP, EP400 and H2A.Z consistently lengthens early mitotic progression. They also detailedly analyzed the LSD1 dataset and performed some experiments to support their findings. Hence, I suggest the authors carefully examine the results of at least one of the datasets in their experiments and explain or discuss whether any possible new biological findings can be found when applying their Time Encoded ResNet18 model.

Reply: Thank you for your comment. We were not able to make any new biological conclusions from the experiments. However, in our experiments with the 6-class dataset, we observed that there is a higher rate of misclassification between adjacent classes in the case of the time-encoded ResNet18 and the misclassification value is quite low for the other classes. This phenomenon comes as an additional advantage stemming from the inclusion of time information. In contrast, for the ResNet18 model without the incorporation of time information, misclassifications are distributed more evenly across all classes. We have added this information now in the paper. Please refer to line numbers 654-659 in the manuscript.

Comment: 4. In the "Conclusions" part, the authors did not discuss the limitations of their model, which are important for users to correctly use their model and avoid possible pitfalls.

Reply: We have updated the 'Conclusions' section of the paper and included the limitations of the paper and future work. Please refer to line numbers 690-702.

Minor points:

Comment: 5. The authors did not provide their codes for this model, making it difficult to try their methods.

Reply: The github link (https://github.com/Rijo756/cell-cycle-stages-identification) is added now. (See the footnote in page 13.).

Comment: 6. In Table 8, the accuracy of LiveCellMiner was higher than the mean accuracy of the Time Encoded ResNet18 model. Therefore, LiveCellMiner should be in boldface rather than Time Encoded ResNet18.

Reply: Thanks for noting this mistake. We have updated Table 8.

Comment: 7. In Table 10 (the analysis of Zhong et al.'s Dataset), I suggest adding the frame-to-frame accuracy of TC3, since it performed the best in predicting cells belonging to the interphase.

Reply: We have referred the TC3 [19] paper for the accuracy numbers. The accuracy value is not reported as a number, but the supplementary file contains a graph showing how the number of features affect the accuracy and the highest value reported for the TC3 model in the paper is 94.1%. We have added that now in the manuscript. Please refer to Table 11.
It is true that the accuracy is slightly lower for the proposed method. Also, please notice that for the pro, prometa, meta, anaphase, and telo-phases the F-score is higher than the TC3 approach. These phases occur much more rarely than the interphase and it is thus great that our method performs better here.

Comment: 8. In the last line on page 3, the names of the authors of reference [26] were missing (after the words "in 2016").

Reply: Thanks for noting this typo. We have updated it. Please check line 113.
